# High content cellular microarray for automated drug target deconvolution

**Published:** 2011-01-10

**Authors:** Yong-Jun Kwon, Hi Chul Kim, Nam Youl Kim, Seo Yeon Choi, Sungyong Jung, Auguste Genovesio

**Affiliations:** 1 Discovery Biology Group, Institut Pasteur Korea, Seongnam-si, 463-400, Korea; 2 Image Mining Group, Institut Pasteur Korea, Seongnam-si, 463-400, Korea

## 

Despite the promising paradigm offered by high-content screening, the concrete execution of hundred of thousands of visual cell-based experiments has remained highly challenging in terms of both statistical robustness and speed. An efficient computational method for cellular microarrays was developed at Institut Pasteur-Korea that allow for high speed, high content genome-wide siRNA screening. Details of the method and examples of data from genome-wide analyses will be featured in this presentation. In particular, we will demonstrate that the sudden ability to dramatically increase the number of experiments has created the opportunity for automated identification of a drug’s target.

